# Exploring the impact of pelvic radiotherapy dose distribution on lymphocyte counts: a voxel-based analysis

**DOI:** 10.1186/s13014-025-02652-5

**Published:** 2025-06-03

**Authors:** Sanghyeok Lee, Seohan Kim, Sangseok Ha, Kyu-Hye Choi, Wonmo Sung

**Affiliations:** 1 Department of Biomedical Engineering and Medical Sciences, College of Medicine, Graduate School of The Catholic University of Korea, Seoul, 137-70 Republic of Korea; 2 Department of Radiation Oncology, Seoul St. Mary’s Hospital, College of Medicine, The Catholic University of Korea, Seoul, 137-70 Republic of Korea; 3 CMC Institute for Basic Medical Science, the Catholic Medical Center of The Catholic University of Korea, Seoul, Republic of Korea

**Keywords:** Radiotherapy, Pelvis, Voxel-based analysis, Radiation-induced lymphopenia, Absolute lymphocyte count

## Abstract

**Background and purpose:**

This study aimed to evaluate the impact of pelvic radiation therapy (RT) on the occurrence of severe radiation-induced lymphopenia (SRIL) and identify its clinical and dosimetric predictors using voxel-wise analysis. Understanding these impacts is crucial for improving patient outcomes and optimizing treatment protocols in radiation oncology.

**Materials and methods:**

A retrospective analysis was conducted on 122 patients who underwent pelvic RT. Absolute lymphocyte counts (ALC) were measured before treatment and within one month of RT initiation. Patients were classified into SRIL and non-SRIL groups on the basis of their lowest recorded ALC during treatment. The associations between SRIL and clinical/dosimetric parameters were assessed via univariable (UVA) and multivariate (MVA) analysis. The influence of regionally detailed dose was assessed by voxel-based analysis (VBA) on spatially normalized 3D dose maps and CT images, focusing on the sacrum, femoral heads, and pelvic bones.

**Results:**

SRIL was associated with clinical and dosimetric factors. The baseline ALC was the most significant clinical predictor, with a lower baseline ALC increasing SRIL risk (OR = 0.996, *p* = 0.001). VBA further revealed localized highly related regional dose patterns, with 92.17% of the left femoral head and 91.32% of the right femoral head showing significant SRIL associations, whereas the associations were significantly lower in the sacrum (10.39%) and pelvic bones (left: 30.01%, right: 31.52%).

**Conclusion:**

This study identified key clinical and dosimetric factors influencing SRIL in patients undergoing pelvic radiotherapy. Baseline ALC was the most significant clinical factor, and VBA showed that regional dose pattern changes within the femoral head were significantly associated with SRIL.

**Clinical trial number:**

Not applicable.

**Supplementary Information:**

The online version contains supplementary material available at 10.1186/s13014-025-02652-5.

## Introduction

Lymphocytes play a crucial role in the immune system, as they are responsible not only for antitumor responses but also for protecting against infections [1, 2]. However, these cells are highly radiosensitive, making them particularly vulnerable to radiation exposure [3, 4]. Radiation-induced lymphopenia (RIL) has been increasingly recognized as a major challenge in radiation therapy (RT), with studies showing that severe lymphocyte depletion leads to higher rates of cancer recurrence, accelerated tumor growth, and diminished effectiveness of immunotherapy [5, 6]. As the integration of immunotherapy into cancer treatment continues to expand, understanding and mitigating RIL is of growing clinical importance [7, 8].

When whole pelvic RT is performed for curative, preoperative, or postoperative purposes for pelvic organ cancer, the The RT field often includes the lower spine and the entire pelvic bone. Additionally, both femur heads are included, which may increase the risk of hematopoietic toxicities, including RIL [9–11]. Thus, it is crucial to quantitatively identify which organs contribute to RIL and to understand their specific dose-response relationships, particularly for integrating this information into pelvic RT planning. However, conventional dosimetric methods may not fully capture the complex spatial variations in radiation sensitivity within these structures.

Voxel-based analysis (VBA) provides a more advanced dosimetric approach compared to conventional methods, as it enables spatially precise dose-response assessments at the voxel level rather than organ level [12, 13]. Traditional organ-based dosimetric methods, such as dose volume histogram (DVH), often depend on averaged dose metrics across an entire organ, which can obscure critical spatial variations in dose-response patterns [14, 15]. In contrast, VBA identifies specific sub-organ regions that exhibit statistically significant associations with clinical endpoints [16]. Given the inherent heterogeneity of anatomical structures and biological functions, accounting for local dose-response variations is essential for improving the prediction of treatment-related effects. For instance, within the pelvic bone, the distribution of lymphatic and vascular networks, as well as the density of bone marrow, varies across different regions, influencing radiation sensitivity [17–19].

This study applies VBA to identify the association between pelvic dose distribution and severe radiation-induced lymphopenia (SRIL). By integrating VBA with conventional logistic regression analysis, it aims to provide a comprehensive understanding of how radiation dose distribution influences SRIL risk.

## Materials & methods

### Population characteristics

This retrospective study included a cohort of 122 patients who underwent pelvic RT at Seoul Saint Mary’s hospital between 2018 and 2022. The treatment was delivered using helical tomotherapy with a conventional fractionation scheme of 1.8–2 Gy per fraction. Dose prescription followed the standard radiotherapy planning approach, with 95% of the prescribed dose covering at least 95% of the target volume. Total treatment duration ranged from 5 to 6 weeks depending on the specific cancer type and treatment protocol. All of the patients were pathologically diagnosed with cancer. The following criteria were met for a patient to be included: (1) had whole pelvic RT of at least 45 Gy; (2) had a Karnofsky performance status score of at least 70; and (3) had complete blood count (CBC) tests performed regularly prior to treatment, once a week during treatment, and within a month after treatment ended. The following patients were excluded from consideration: (1) those who received chemotherapy within three months of RT; (2) those who had RT interrupted for more than one week for any reason; (3) those who had secondary primary malignancies; and (4) those with an absolute lymphocyte count (ALC) of ≥ grade 1 (≥ 800 #/µL) at the first measurement.

Informed consent was waived because of the retrospective nature of the study, and approval from the Institutional Review Board (IRB) was obtained prior to the commencement of the study (IRB No. KC22RISI0715). Patients were divided into two groups, the SRIL group and the non-SRIL group, on the basis of their lowest ALC during treatment. For each patient, the ALC was obtained through a CBC test during treatment. Lymphocyte toxicity was categorized on the basis of the Common Terminology Criteria for Adverse Events (CTCAE v5.0). ALC was categorized into four grades: Grade 1 (-800/L), Grade 2 (500–800/L), Grade 3 (200–500/L), and Grade 4 (≤ 200/L). ALC with less than 500 cells/µL was defined as SRIL. There were 26 people in the SRIL group and 96 people in the non-SRIL group (Table 1).

**Table 1:** Patient characteristics

Variable	Non SRIL (*n* = 96)	SRIL (*n* = 26)	Total (*N* = 122)
Sex, no. (%)			
Male	81 (84.38)	15 (57.69)	96 (78.7)
Female	15 (15.62)	11 (42.31)	26 (21.3)
Age, no. (%)			
68 ≤	45 (46.88)	18 (69.23)	63 (51.64)
68 >	51 (53.12)	8 (30.77)	59 (48.36)
Cancer type, no. (%)			
Bladder	1 (1)	2 (7.7)	3 (2.5)
Prostate	80 (83.3)	13 (50)	93 (76.2)
Cervix	5 (5.2)	6 (23.1)	11 (9)
Endometrium	10 (10.4)	5 (19.2)	15 (12.3)
Lymphopenia grade, no. (%)			
0	9 (9.4)		9 (7.4)
1	22 (22.9)		22 (18)
2	65 (67.7)		65 (53.3)
3		26 (100)	26 (21.3)
Baseline ALC, median (range), /µL	1389.72 (960.4-1972.4)	1045.68 (909.4-1793.5)	1330.56 (1030.5-1972.4)
Lowest ALC on RT, median (range), /µL	728.15 (500.6-1388.1)	379.54 (209.9-470.9)	660.72 (209.9-1388.1)
Fraction at lowest ALC, median (range), no.	20 (14–28)	23 (18–27)	21 (14–28)
Total dose at lowest ALC^*^, median (range), cGy	4750 (3240–7000)	4750 (3780–5750)	4750 (3240–7000)
V_**10 Gy**_, median (range), volume,%			
Left femoral head	49.05 (0.75–94.07)	63.78 (23.47–89.61)	52.12 (0.75–94.07)
Right femoral head	50.25 (1.22–88.54)	61.22 (28.84–96.39)	52.27 (1.22–96.39)
Left pelvic bone	80.32 (22.84–95.07)	83.28 (62.53–96.87)	80.69 (22.84–96.87)
Right pelvic bone	83.02 (22.67–95.52)	85.79 (67.28–97.78)	83.31 (22.67–97.78)
Sacrum	95.06 (1.18–100.00)	98.44 (43.95–100.00)	95.37 (1.18–100.00)
V_**20 Gy**_, median (range), volume,%			
Left femoral head	3.87 (0.00–30.10)	7.13 (0.00–27.89)	4.04 (0.00–30.10)
Right femoral head	6.88 (0.00–30.18)	10.71 (0.00–29.97)	7.32 (0.00–30.18)
Left pelvic bone	46.98 (4.87–74.13)	51.17 (22.90–74.68)	47.48 (4.87–74.68)
Right pelvic bone	48.78 (3.84–78.69)	52.72 (17.32–87.39)	49.24 (3.84–87.39)
Sacrum	78.34 (0.00–99.95)	86.70 (35.86–100.00)	80.19 (0.00–100.00)
V_**30 Gy**_, median (range), volume,%			
Left femoral head	0.00 (0.00–5.07)	0.01 (0.00–5.49)	0.00 (0.00–5.49)
Right femoral head	0.00 (0.00–5.13)	0.01 (0.00–5.84)	0.00 (0.00–5.84)
Left pelvic bone	12.04 (0.25–45.70)	15.85 (1.12–50.64)	12.96 (0.25–50.64)
Right pelvic bone	15.01 (0.46–45.25)	19.63 (1.86–62.30)	15.59 (0.46–62.30)
Sacrum	36.88 (0.00–79.26)	45.37 (2.43–99.85)	39.41 (0.00–99.85)
V_**40 Gy**_, median (range), volume,%			
Left femoral head	0.00 (0.00–0.12)	0.00 (0.00–0.01)	0.00 (0.00–0.12)
Right femoral head	0.00 (0.00–0.07)	0.00 (0.00–0.02)	0.00 (0.00–0.07)
Left pelvic bone	1.48 (0.00–21.57)	2.53 (0.00–20.31)	1.75 (0.00–21.57)
Right pelvic bone	2.32 (0.00–17.47)	4.07 (0.00–27.33)	2.90 (0.00–27.33)
Sacrum	0.00 (0.00–51.65)	6.90 (0.00–65.21)	0.00 (0.00–65.21)
Mean dose, mean ± SD, Gy			
Left femoral head	10.45 ± 2.23	11.90 ± 2.43	10.76 ± 2.34
Right femoral head	10.88 ± 2.30	12.43 ± 2.56	11.21 ± 2.43
Left pelvic bone	18.61 ± 3.64	20.25 ± 3.54	18.96 ± 3.67
Right pelvic bone	19.46 ± 3.65	20.95 ± 3.97	19.78 ± 3.75
Sacrum	25.56 ± 5.27	28.29 ± 6.68	26.14 ± 5.68

Lymphopenia grade was determined at the time of lowest ALC count during RT

Abbreviations: ALC = absolute lymphocytes count; RT = radiation therapy; SRIL = severe radiation induced lymphopenia; SD = standard deviation

Total dose at lowest ALC^*^ = Treatment plan dose received by the target volume at the time point at which the lowest ALC was measured

### Registration

The dose calculation grid was set to 1 mm × 1 mm × 1 mm. All CT and dose images were resampled to a uniform voxel size of 1 mm × 1 mm × 3 mm, which provided a balance between anatomical detail and computational efficiency. This voxel size was maintained throughout the analysis to ensure consistency and reduce interpolation artifacts during deformation and dose warping. The CT scans were first cropped to focus on the pelvic region (from 30 mm above the iliac crest to 30 mm below the femoral head), and the window level/width was set to 500 and 2000, respectively, to enhance contrast. Pelvic structures including the sacrum, femoral heads, and pelvic bones were automatically segmented using Manteia software and converted into binary masks.

Rigid registration was performed to achieve initial alignment based on anatomical landmarks, followed by deformable registration using a B-spline algorithm implemented in Plastimatch [20]. The reference patient was selected based on median pelvic length (cranio-caudal axis), as this dimension reflects the primary treatment field in pelvic radiotherapy and allows for consistent alignment across the cohort.

To ensure the quality of deformable registration, we evaluated root mean square error (RMSE), Dice similarity coefficient (DSC), and relative difference in area (RDA) before and after registration. Patients were considered to have passed registration if these metrics improved or remained within an acceptable range following deformation (Table S1).

### Statistical analysis

The influence of clinical factors on SRIL was analyzed via UVA and MVA using R (version 4.3.2). UVA was used to assess the individual effect of each factor, whereas MVA accounted for potential confounding variables to ensure a more comprehensive evaluation. The factors included in the analysis were sex and age (categorized on the basis of the median value), baseline ALC, the fraction at which the lowest ALC was measured, the total radiation dose at the time of lowest ALC, V_**10 Gy**_-V_**40 Gy**_ (Volume percentages of a structure receiving at least 10 to 40 Gy of radiation) and the mean radiation dose (bone structure). This study defines the clinical endpoint as the time at which the lowest ALC was recorded. In order to match the timeline, all dose-volume parameters were recalculated by proportionally adjusting the total dose according to the number of fractions delivered up to that nadir.

**Fig. 1:**
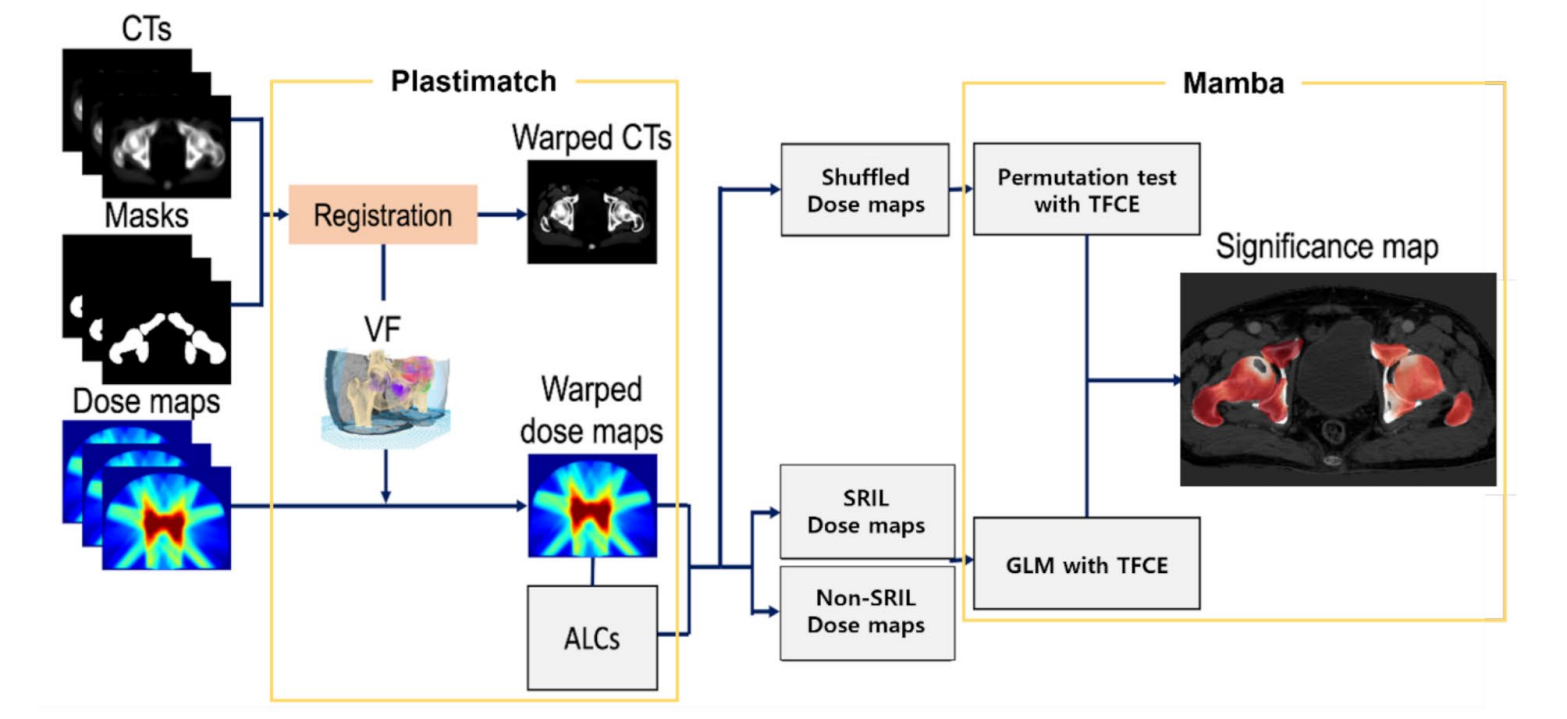
Workflow of the study. A VBA involves two primary steps. First, the diverse anatomical structures within the analyzed patient cohort were spatially normalized to a common coordinate system. After the RT dose maps are aligned for precise voxel-by-voxel comparisons, statistical inference is applied to identify regions where significant correlations exist between clinical outcomes and local dose distributions, revealing the spatial patterns of the dose response. Abbreviations CT = computed tomography

To further investigate significant voxelwise differences in radiation dose distributions between the SRIL and non-SRIL cohorts, VBA was performed via the MultipAradigM voxel-Based Analysis (MAMBA) toolkit [14, 15]. The registered dose maps were imported into MAMBA for analysis (Fig. 1), providing a detailed spatial assessment of radiation dose variations.

A Generalized linear model (GLM) was used to assess the association between SRIL and radiation dose at each voxel. Factors that showed significant associations in the MVA results were selected as correction coefficients.

A non-parametric permutation test was performed with 1,000 iterations, during which the SRIL and non-SRIL group labels were randomly reassigned while maintaining the original group sizes. For each permutation, the maximum voxel-wise statistic was recorded to control the family-wise error. Statistical significance was determined by comparing the observed test statistics to the empirical null distribution generated through these permutations. To enhance statistical robustness, Threshold-Free Cluster Enhancement (TFCE) [21] was applied to the voxel-wise *p*-values. TFCE improves the detection of spatially contiguous regions by enhancing voxel clusters without relying on an arbitrary threshold, thereby enabling more reliable identification of significant areas. Regions were considered statistically significant if the TFCE adjusted *p*-value was less than 0.05.

## Results

### Population characteristics

This study analyzed data from 122 patients diagnosed with bladder, prostate, cervical, or endometrial cancer who underwent pelvic RT between 2018 and 2022; the detailed patient characteristics are summarized in Table 1. The baseline ALC was measured either before or within one week of RT initiation, and the lowest ALC recorded during RT was used to classify patients into five lymphopenia grades. Among the participants, 96 patients (78.7%) experienced RILs, whereas 26 patients (21.3%) developed SRIL (Table 1).

### Statistical analysis

**Fig. 2:**
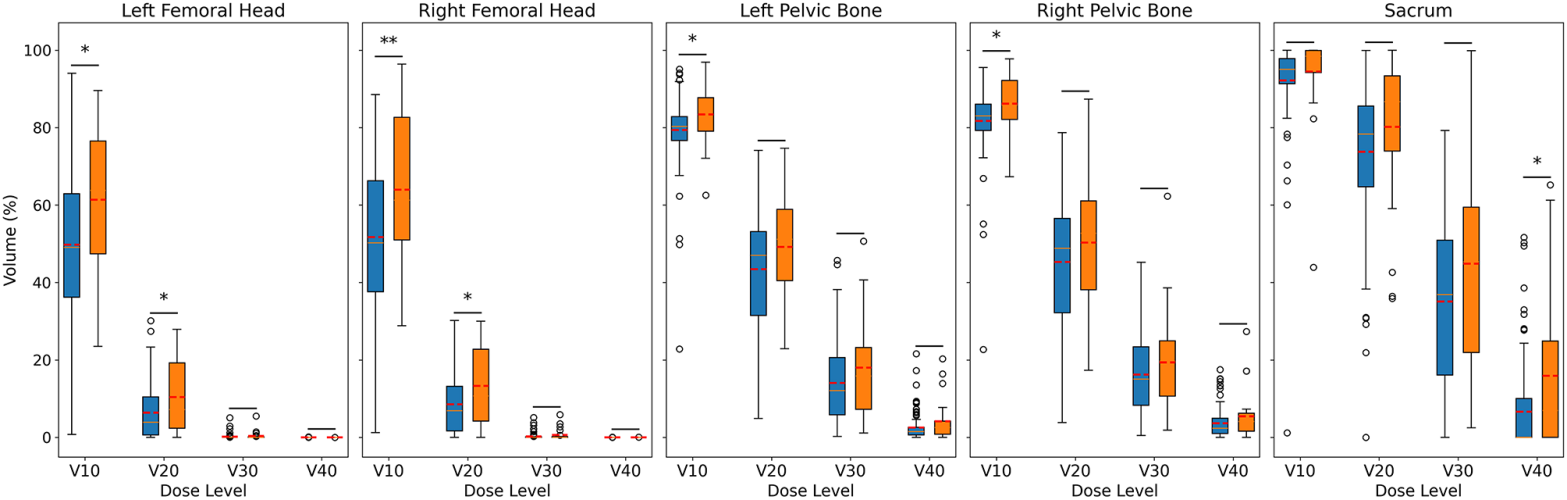
Comparison of V_10 Gy_–V_40 Gy_ (%) values for structures between Non-SRIL and SRIL groups. Each subplot represents a different anatomical region: Left Femoral Head, Right Femoral Head, Left Pelvic Bone, Right Pelvic Bone, and Sacrum. Blue boxes represent the Non-SRIL group, and orange boxes represent the SRIL group. Red horizontal lines indicate group means. Statistical significance between groups was assessed using Welch’s t-test. * *p* < 0.05, ** *p* < 0.01, *** *p* < 0.001

Compared with the non-SRIL group, the SRIL group presented greater radiation exposure across all evaluated pelvic structures, particularly at lower dose levels. At V_**10 Gy**_, right femoral head (87.6% vs. 57.9%, *p* = 0.003), left femoral head (84.8% vs. 63.4%, *p* = 0.003), right pelvic bone (90.2% vs. 78.7%, *p* = 0.010), and left pelvic bone (86.4% vs. 74.1%, *p* = 0.006). Although the sacrum had a high V_**10 Gy**_ in both groups, the difference was not statistically significant (97.3% vs. 94.1%, *p* = 0.353). At the V_**20 Gy**_ level, the SRIL group continued to exhibit greater irradiated volumes, with significant differences in the right femoral head (12.3% vs. 4.9%, *p* = 0.049), and borderline significance observed in the left femoral head (10.6% vs. 5.1%, *p* = 0.055) and sacrum (63.5% vs. 52.1%, *p* = 0.066). No significant differences were found in the pelvic bones at this dose level. At V_**30 Gy**_ and V_**40 Gy**_, the differences between groups were less pronounced, with only the sacrum showing a statistically significant difference at V_**40 Gy**_ (31.2% vs. 19.8%, *p* = 0.017), while other structures remained comparable between groups (Fig. 2).

**Fig. 3:**
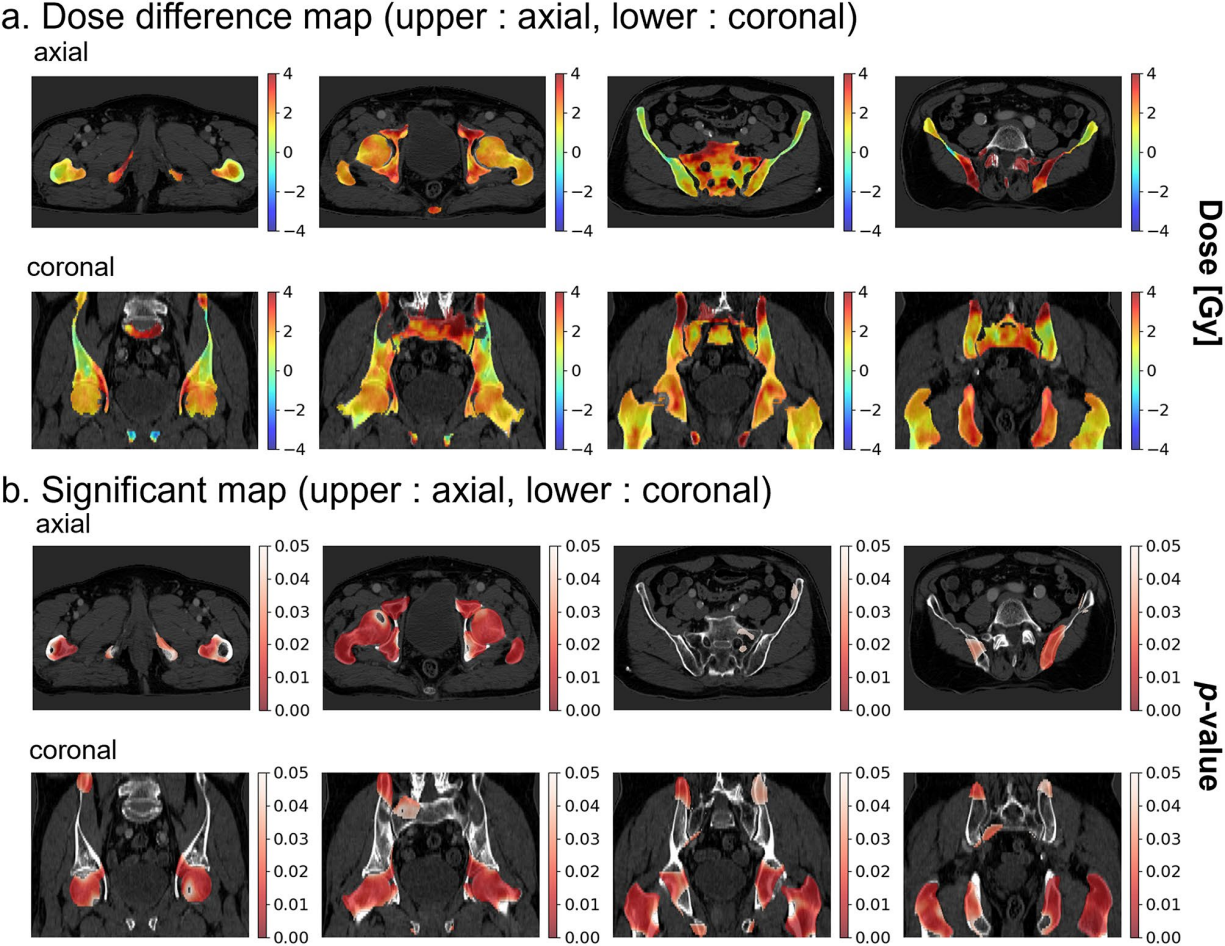
Dose difference map and significance map. (**a**) Differences in the doses received by the SRIL group and the non-SRIL group. It is calculated by subtracting the dose map of the non-SRIL group from the dose map of the SRIL group. (**b**) Visualizes significant *p* values. The darker the red color is, the greater the correlation with the occurrence of SIRL

These volume-based findings were consistent with mean dose comparisons, which demonstrated that the SRIL group received higher average doses across all structures: mean doses of 28.29 ± 6.68 Gy vs. 25.56 ± 5.27 Gy in the sacrum, mean doses of 11.90 ± 2.43 Gy vs. 10.45 ± 2.23 Gy in the left femoral head, mean doses of 12.43 ± 2.56 Gy vs. 10.88 ± 2.30 Gy in the right femoral head, mean doses of 20.25 ± 3.54 Gy vs. 18.61 ± 3.64 Gy in the left pelvic bone, and mean doses of 20.95 ± 3.97 Gy vs. 19.46 ± 3.65 Gy in the right pelvic bone (Table 1). VBA revealed statistically significant dose differences between the SRIL and non-SRIL groups. Figure 3a shows the dose difference map obtained by subtracting the mean dose map of the non-SRIL group from that of the SRIL group. Figure 3b presents the significance map, where *p* values are transformed into a -log (*p* value) format to highlight significant dose‒SRIL associations.

The association between radiation dose parameters and the development of SRIL was assessed through both MVA and VBA. In the MVA, lower baseline ALC remained a strong and independent predictor of SRIL (OR 0.996; 95% CI, 0.994–0.998; *p* = 0.001) (Table 2). While higher V_**10 Gy**_ and V_**20 Gy**_ dose–volume parameters to the femoral heads and pelvic bones were significantly associated with SRIL in UVA, these associations did not remain statistically significant in the MVA. Mean doses to the femoral heads, pelvic bones, and sacrum also showed significant associations in UVA, however none persisted after adjusting for clinical covariates, suggesting possible confounding effects.

**Table 2:** Univariable/Multivariate analysis for factors associated with SRIL

	Univariable analysis						Multivariate analysis						
			95% CI						95% CI				
Variable	Odds ratio	Lower		Upper	Unit	*P* value	Odds ratio	Lower		Upper	Unit	*P* value	
Baseline ALC	0.996	0.994		0.998	Counts*µL^− 1^	< 0.001	0.996	0.994		0.998	Counts*µL-1	0.001	
Fraction at lowest ALC	1.302	1.109		1.557	Fractions^− 1^	0.002	1.333	1.002		1.857	Fractions-1	0.065	
Total dose at lowest ALC^*^	1.000	0.999		1.001	Gy^− 1^	0.951					Gy^− 1^		
Sex, (male vs., female)	3.960	1.526		10.27	Unitless	0.005	0.576	0.075		3.529	Unitless	0.571	
Age	0.941	0.899		0.985	Years^− 1^	0.009	0.961	0.902		1.019	Years-1	0.195	
V_**10 Gy**_													
Left femoral head	1.033	1.009		1.06	Percentage^− 1^	0.01	0.958	0.812		1.123	Percentage^− 1^	0.597	
Right femoral head	1.036	1.011		1.063	Percentage^− 1^	0.006	1.064	0.892		1.284	Percentage^− 1^	0.502	
Left pelvic bone	1.086	1.014		1.173	Percentage^− 1^	0.029	1.205	0.926		1.578	Percentage^− 1^	0.159	
Right pelvic bone	1.109	1.028		1.207	Percentage^− 1^	0.012	0.957	0.743		1.233	Percentage^− 1^	0.73	
Sacrum	1.026	0.982		1.105	Percentage^− 1^	0.386					Percentage^− 1^		
V_**20 Gy**_													
Left femoral head	1.065	1.009		1.124	Percentage^− 1^	0.021	1.078	0.784		1.504	Percentage^− 1^	0.646	
Right femoral head	1.062	1.012		1.116	Percentage^− 1^	0.016	0.933	0.702		1.232	Percentage^− 1^	0.625	
Left pelvic bone	1.025	0.996		1.058	Percentage^− 1^	0.102					Percentage^− 1^		
Right pelvic bone	1.019	0.992		1.049	Percentage^− 1^	0.172					Percentage^− 1^		
Sacrum	1.022	0.996		1.054	Percentage^− 1^	0.126					Percentage^− 1^		
V_**30 Gy**_													
Left femoral head	1.321	0.783		2.233	Percentage^− 1^	0.256					Percentage^− 1^		
Right femoral head	1.343	0.892		2.021	Percentage^− 1^	0.139					Percentage^− 1^		
Left pelvic bone	1.032	0.993		1.072	Percentage^− 1^	0.105					Percentage^− 1^		
Right pelvic bone	1.025	0.986		1.064	Percentage^− 1^	0.204					Percentage^− 1^		
Sacrum	1.018	0.999		1.038	Percentage^− 1^	0.066					Percentage^− 1^		
V_**40 Gy**_													
Left femoral head	NA	NA		NA	Percentage^− 1^	NA					Percentage^− 1^		
Right femoral head	NA	NA		NA	Percentage^− 1^	NA					Percentage^− 1^		
Left pelvic bone	1.088	0.986		1.201	Percentage^− 1^	0.084					Percentage^− 1^		
Right pelvic bone	1.08	0.989		1.182	Percentage^− 1^	0.082					Percentage^− 1^		
Sacrum	1.036	1.009		1.065	Percentage^− 1^	0.008	1.037	0.96		1.122	Percentage^− 1^	0.36	
Mean dose													
Left femoral head	1.317	1.086		1.625	Gy^− 1^	0.007	1.151	0.236		6.07	Gy-1	0.863	
Right femoral head	1.319	1.093		1.62	Gy^− 1^	0.005	0.952	0.17		5.011	Gy-1	0.954	
Left pelvic bone	1.141	1.007		1.305	Gy^− 1^	0.044	0.658	0.381		1.063	Gy-1	0.105	
Right pelvic bone	1.12	0.993		1.276	Gy^− 1^	0.074					Gy^− 1^		
Sacrum	1.098	1.012		1.201	Gy^− 1^	0.031	0.954	0.736		1.237	Gy-1	0.72	

MVA was performed with the variables that showed *p*-value lower than 0.05 in UVA. Volume receiving higher than 40 Gy of left and right femoral head were not included in the analysis owing to substantial missing data

Abbreviations: CI = confidence interval; ALC = absolute lymphocyte counts; RT = radiation therapy; RIL = radiation induced lymphopenia; Total dose at lowest ALC^*^ = Treatment plan dose received by the target volume at the time point at which the lowest ALC was measured

**Fig. 4:**
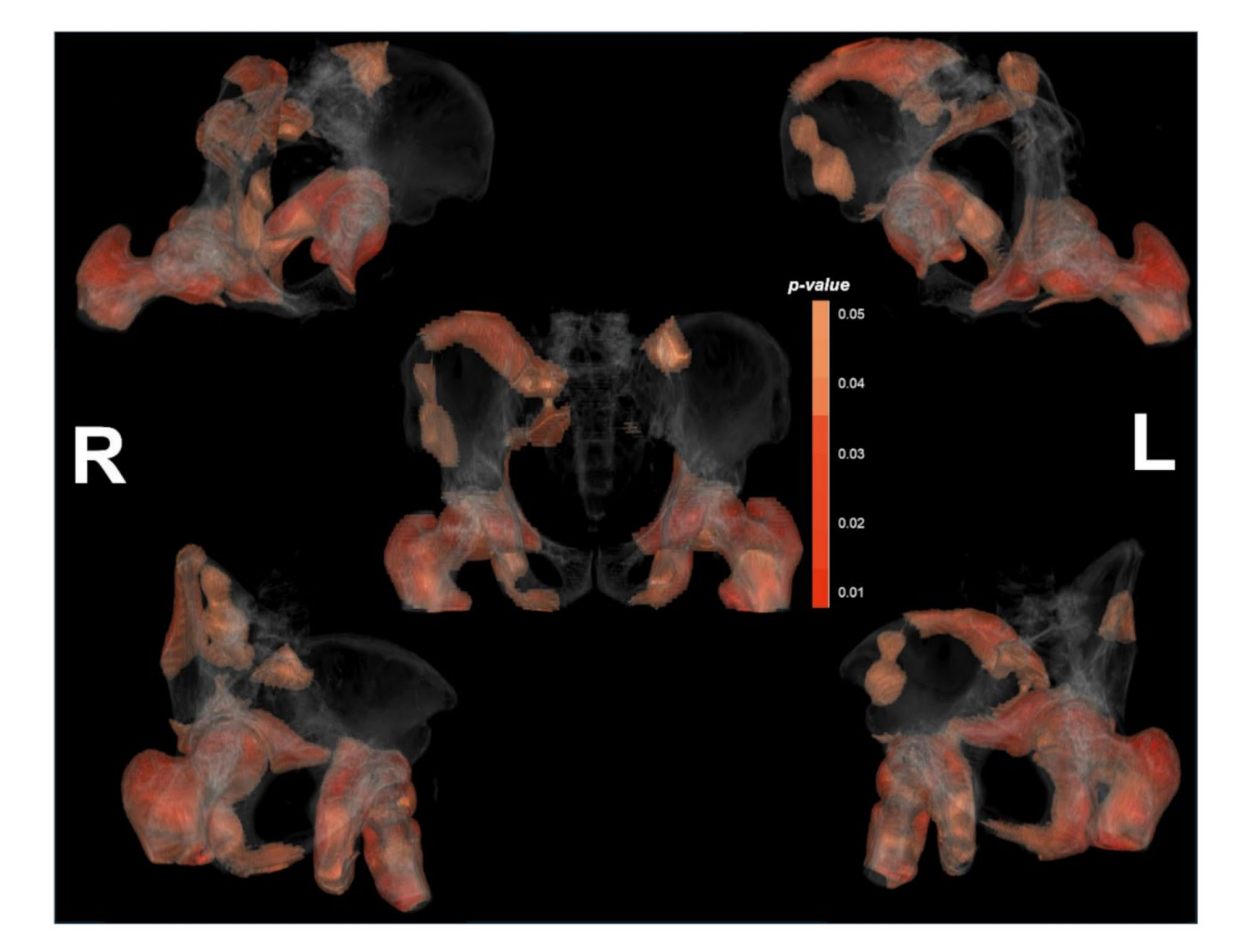
3D significance map. The CT image was reconstructed in 3D via the 3D rendering function in 3D slicer, and the *p* values for each voxel obtained via the MAMBA toolkit and expressed by matching it to the color bar on the figure. Finally, the two images are combined on a 3D slicer to obtain the corresponding figure

**Fig. 5:**
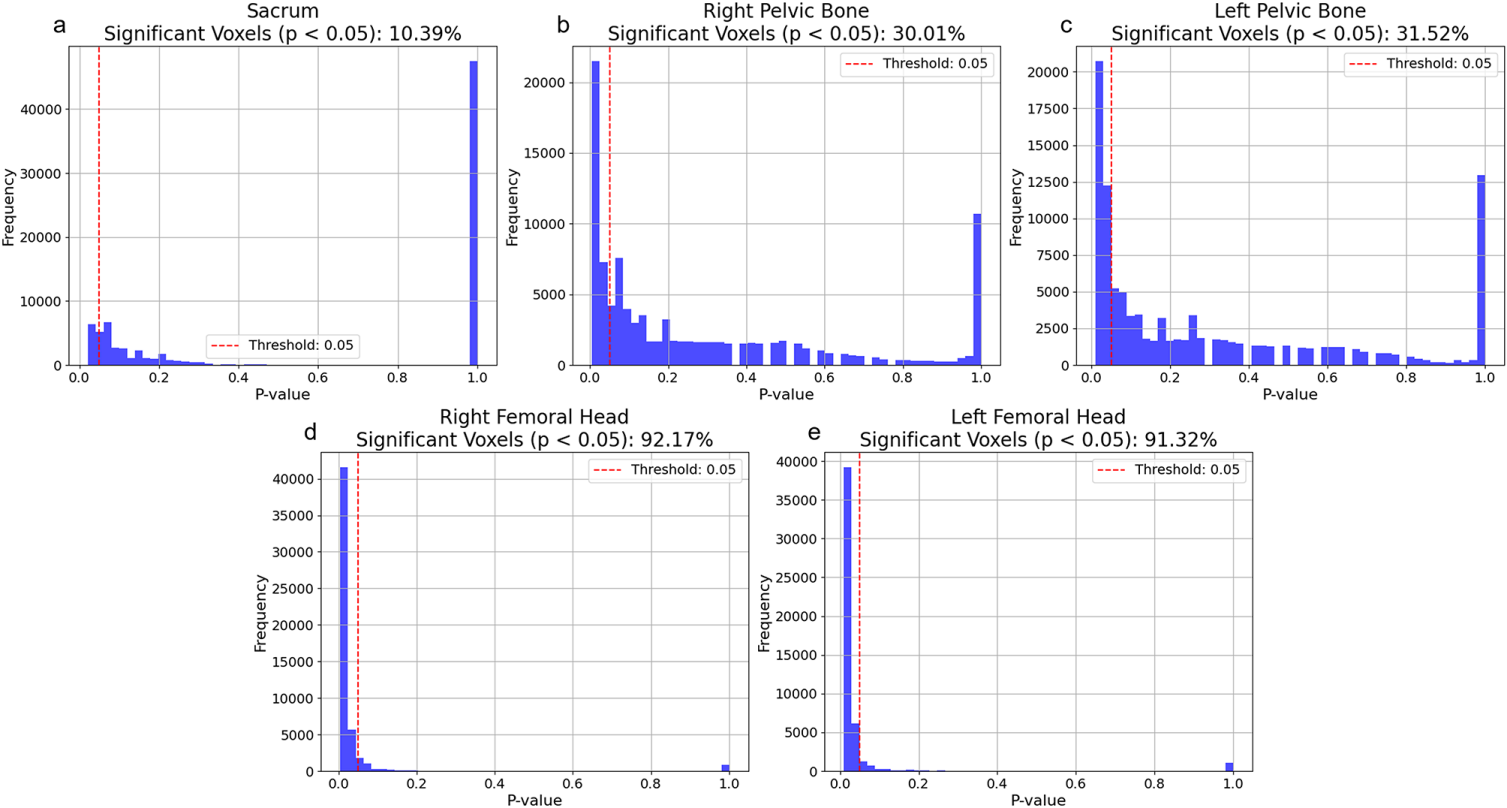
Histogram for significant areas in each segmented region. (**a**. Sacrum 10.39%, **b**. Right Pelvic Bone 30.01%, **c**. Left Pelvic Bone 31.52%, **d**. Left Femoral Head 91.32%, **e**. Right Femoral Head 92.17%) Counts number of significant voxels in each segmented region. Here, significant voxels have lower *p*-value than 0.05

To further explore spatial dose–effect relationships, VBA was conducted to identify voxel level regions significantly associated with SRIL. The results of the quantitative analysis of significant dose regions are shown in Fig. 4. The femoral heads represented the largest proportion of significant dose regions, with 92.17% of the left femoral head and 91.32% of the right femoral head demonstrating strong associations with SRIL. In contrast, the sacrum (10.39%) and pelvic bones (left: 30.01%, right: 31.52%) exhibited lower proportions of SRIL associated voxels (Fig. 5). These findings suggest that both systemic factors such as baseline ALC and localized high dose sensitivity region to the development of SRIL. Notably, the femoral heads, despite their relatively lower marrow content, emerged as critical dose-sensitive regions, underscoring the importance of spatial dose distribution in SRIL risk assessment.

## Discussion

This study emphasizes the significant association between baseline ALC and radiation dose to pelvic bone structures in the development of SRIL among patients undergoing pelvic RT. The integration of both VBA and logistic regression analysis provides complementary insights into the spatial and statistical relationships between local dose distributions and SRIL occurrence. Although UVA showed that the sacrum mean dose (OR = 1.137, *p* = 0.031) was significantly associated with SRIL, this association was not retained in the MVA. The sacrum V_**10 Gy**_ was included in the MVA however was not statistically significant (OR = 1.037, *p* = 0.36), indicating that the sacrum did not independently predict SRIL when adjusted for other factors.

Interestingly, some structures such as the femoral heads, pelvic bones, and even the sacrum showed statistical significance in the UVA, including the right femoral head (mean dose OR = 1.319, *p* = 0.005), left femoral head (OR = 1.317, *p* = 0.007), and left pelvic bone (OR = 1.141, *p* = 0.044). However, these associations were not retained in the MVA, where only baseline ALC (OR = 0.996, 95% CI: 0.994–0.998, *p* = 0.001) and sacrum V_**10 Gy**_ (OR = 1.037, *p* = 0.36) were evaluated, and no dosimetric variable remained statistically significant.

Notably, despite their loss of significance in MVA, the femoral heads were highly significant in VBA, with 92.17% (left) and 91.32% (right) of their volumes exceeding the significance threshold. We applied both t-tests and GLM using the MAMBA toolkit. While the t-tests showed no significant group differences, the GLM revealed significance only when baseline ALC, which had been identified as a significant factor in the MVA, was included as a covariate.

Our findings suggest that subregions of the pelvic bone marrow may vary in their radiosensitivity [9, 11, 22, 23]. This conclusion is based on the observation that only specific structures, such as the femoral head, were significantly associated with SRIL, even though they contain less red marrow compared to regions like the sacrum [24]. Although these results are suggestive, further biological validation, such as bone marrow activity imaging or cell tracking studies, is needed to confirm differences in radiosensitivity across pelvic bone marrow regions.

Several studies have reported that hematologic toxicity correlates with the irradiated volume of pelvic bone at specific dose levels. For example, pelvic bone V_**20 Gy**_ has been associated with ≥ Grade 2 hematologic toxicity, while both V_**10 Gy**_ and V_**20 Gy**_ have been linked to ≥ Grade 3 toxicity in patients treated with IMRT [25–27]. These findings emphasize the relevance of dose-volume parameters in predicting hematologic toxicity. However, MVA, which often relies on organ-level mean dose values, may fail to capture the immunologic consequences of spatial heterogeneity within irradiated bone. This is particularly important because localized high-dose sensitivity subregions may disproportionately contribute to lymphocyte depletion, even if the mean dose remains within acceptable limits. VBA addresses this limitation by enabling three-dimensional voxel-wise analysis of dose distribution, thereby identifying dose-sensitive areas that are significantly associated with SRIL [13, 16, 28].

This study has several limitations. First, the analysis was restricted to bone structures and did not account for lymphoid components such as pelvic lymph nodes. Major lymph node groups, including the inguinal and iliac nodes, are located within the pelvis and play a critical role in lymphocyte trafficking and immune regulation [29]. Therefore, future studies should incorporate both lymphoid and vascular structures to more comprehensively evaluate radiation-induced immunosuppression in the pelvic region.

Second, there is a lack of biological assays or functional imaging to directly assess bone marrow activation. However, recent studies have described potential treatment planning strategies that use fused MRI or PET images to identify red bone marrow for dose evaluation [30]. Additional studies that include biologically quantified dose-risk data for hematopoietic and lymphoid organs such as the above are needed to support the clinical application of the study results.

Finally, inter-fraction motion may introduce uncertainty in voxel-wise dose comparisons. Radiation therapy is delivered across multiple fractions, and despite patient immobilization, variations in organ position and patient condition can cause discrepancies between the planned and delivered dose [31, 32]. Such variations may blur the spatial boundaries of statistically significant regions in VBA or even prevent accurate identification of true dose sensitive areas.

In summary, this study underscores the critical role of both baseline lymphocyte status and spatial radiation dose distribution in the development of SRIL among patients receiving pelvic radiotherapy. While traditional statistical approaches such as logistic regression highlight the importance of baseline ALC as a dominant predictor, voxel-based analysis reveals that specific subregions within the pelvic bone particularly the femoral heads may contribute significantly to immunosuppression despite not being retained in multivariable models. These findings suggest that reliance on organ-level mean dose metrics may underestimate localized dose effects on immune function. By integrating spatial and statistical methodologies, our approach provides a more nuanced understanding of radiation-induced lymphopenia and highlights the need for personalized treatment strategies that minimize radiation exposure to immunologically active bone regions. Building on our previous work involving NTCP modeling for SRIL [33], the clinical applicability of this study could be further enhanced by incorporating additional factors such as lymphoid structures and biological markers of myeloid activity.

## Conclusion

This study provides new insights into clinical and dose predictors of SRIL in patients receiving pelvic radiotherapy. Baseline ALC was the strongest clinical predictor of SRIL (OR = 0.996, *p* < 0.001). Dosimetrically, MVA did not maintain statistical significance for any structure, but the VBA showed that spatial dose patterns change within the femoral head were significantly associated with SRIL, with more than 90% of both femoral heads showing voxel level significance.

These results highlight the unique value of VBA regional dose patterns may contribute to SRIL risk that may be overlooked by traditional DVH indices.

## Electronic supplementary material

Below is the link to the electronic supplementary material.

Supplementary Material 1

## Data Availability

No datasets were generated or analysed during the current study.

## Abbreviations

RT: Radiotherapy
RIL: Radiation induced lymphopenia
SRIL: Severe radiation induced lymphopenia
ALC: Absolute lymphocytes counts
UVA: Univariable analysis
MVA: Multivariate analysis
VBA: Voxel-based analysis
OR: Odds ratio
DVH: Dose volume histogram
CBC: Complete blood count
IRB: Institutional review board
CCS: Common coordinate system
RMSE: Root mean square error
DSC: Dice similarity coefficient
RDA: Relative difference of areas
DOO: Dose‒organ overlap
MAMBA: MultipAradigM voxel-based analysis
GLM: Generalized linear model
TFCE: Threshold-free cluster enhancement
SD: Standard deviation
NTCP: Normal tissue complication probability
