# Supplementary material for: Exploring the impact of pelvic radiotherapy dose distribution on lymphocyte counts: a voxel-based analysis
Source: Radiat Oncol. 2025 Jun 3;20:92. doi: 10.1186/s13014-025-02652-5 (PMC12135238; doi:10.1186/s13014-025-02652-5)
Supplement: Supplementary file 1 — Supplementary Material 1 [file 13014_2025_2652_MOESM1_ESM.docx]

Supplementary Table 1. Registration evaluation value table

The table compares quantitative indicators such as root mean square error (RMSE), dice similarity coefficient (DSC), relative difference of areas (RDA), and dose organ overlap (DOO) before and after registration for different patients, indicating improvements in registration accuracy. For RMSE, if the after value is smaller than the before value, it is considered a pass. For DSC, RDA, and DOO, if the after value is larger than the before value, it is considered a pass. All patient data that used in this study shown improved value before registration.

| **Variable** | **Median (range)** | **Pass or Fail** |
| --- | --- | --- |
| **rmse_before** | 79.534 (70.735-91.731) | Pass |
| **rmse_after** | 29.493 (25.976-37.565) |  |
| **dsc_before** | 0.7125 (0.604-0.788) | Pass |
| **dsc_after** | 0.7865 (0.725-0.827) |  |
| **rda_before** | 0.07 (0.017-0.288) | Pass |
| **rda_after** | 0.287 (0.222-0.361) |  |
| **doo_before** | 0.5955 (0.471-0.702) | Pass |
| **doo_after** | 0.658 (0.601-0.71) |  |
| **Abbreviations: rmse = root mean square error;  dsc = dice similarity coefficient;  rda = relative difference of areas;  doo = dose organ overlap;** | | |
